# Association rules for rat spatial learning: The importance of the hippocampus for binding item identity with item location

**DOI:** 10.1002/hipo.22154

**Published:** 2013-07-10

**Authors:** Mathieu M Albasser, Julie R Dumont, Eman Amin, Joshua D Holmes, Murray R Horne, John M Pearce, John P Aggleton

**Affiliations:** 1 School of Psychology, Cardiff UniversityCardiff, Wales, United Kingdom; 2 Horne is currently at Laboratory of Neurobiology and Cognition, CNRS, University de Provence13331, Marseille cedex 3, France

**Keywords:** associative memory, biconditional learning, configural learning, hippocampal formation, spatial memory

## Abstract

Three cohorts of rats with extensive hippocampal lesions received multiple tests to examine the relationships between particular forms of associative learning and an influential account of hippocampal function (the cognitive map hypothesis). Hippocampal lesions spared both the ability to discriminate two different digging media and to discriminate two different room locations in a go/no-go task when each location was approached from a single direction. Hippocampal lesions had, however, differential effects on a more complex task (biconditional discrimination) where the correct response was signaled by the presence or absence of specific cues. For all biconditional tasks, digging in one medium (A) was rewarded in the presence of cue C, while digging in medium B was rewarded in the presences of cue D. Such biconditional tasks are “configural” as no individual cue or element predicts the solution (AC+, AD−, BD+, and BC−). When proximal context cues signaled the correct digging choice, biconditional learning was seemingly unaffected by hippocampal lesions. Severe deficits occurred, however, when the correct digging choice was signaled by distal room cues. Also, impaired was the ability to discriminate two locations when each location was approached from two directions. A task demand that predicted those tasks impaired by hippocampal damage was the need to combine specific cues with their relative spatial positions (“structural learning”). This ability makes it possible to distinguish the same cues set in different spatial arrays. Thus, the hippocampus appears necessary for configural discriminations involving structure, discriminations that potentially underlie the creation of cognitive maps.

## INTRODUCTION

The “cognitive map” hypothesis (O'Keefe and Nadel, 1978) is one of the most influential models of rodent hippocampal function. In this model, the hippocampus helps construct spatial representations using the relative positions of multiple, distal stimuli. The resulting cognitive map is global as it is largely independent of specific viewpoints or directions of travel. Extensive support comes from electrophysiological studies of “place cells” (O'Keefe, 1979; O'Keefe and Burgess, 1996; Moser et al., 2008) and from hippocampal lesion studies. A classic example is that hippocampal lesions disrupt location learning in the Morris water maze when determined by the relative positions of extra-maze cues (Morris et al., 1982). In contrast, rats with hippocampal lesions can solve location problems in the Morris maze by reference to a single, salient cue (Pearce et al., 1998).

As an addition to the hippocampus providing a cognitive map, it has been proposed that the hippocampus is more generally required for “configural” or “relational” learning (Sutherland and Rudy, 1989; Rudy and Sutherland, 1995; Eichenbaum, 2004). The configural learning hypothesis predicts that hippocampal damage will spare tasks solved by learning about single cues (elements), but impair tasks that demand the conjunction of cues for their solution (non-elemental or configural learning). Examples of the latter include biconditional learning. In a biconditional task there are four elements (A, B, C, and D), and no individual element predicts the solution. Rather, each element pairing has a particular outcome, for example, AC+, AD−, BD+, and BC−. From this standpoint, the disruptive influence of hippocampal lesions on cognitive maps is attributed to an inability to form stimulus configurations, deemed essential if one location is to be distinguished from another. Indeed, it is the ability to form stimulus configurations that potentially underlies the global nature of the cognitive map. The configural learning hypothesis has, however, received only mixed support from hippocampal lesion studies (e.g., Gallagher and Holland, 1992; Jarrard, 1993; McDonald et al., 1997; Sanderson et al., 2006). The present experiments, therefore, examined why only some configural tasks are sensitive to hippocampal damage by testing the link between configural and spatial learning.

The study was centered around spatial biconditional learning, a configural task dependent on the hippocampus (e.g., Gaffan and Harrison, 1989; Sziklas et al., 1998; Deacon et al., 2001; Sziklas and Petrides, 2002; Henry et al., 2004; Dumont et al., 2007). The present version involved searching for food in medium A in condition C but not condition D, and searching for food in medium B in condition D but not condition C (i.e., AC+, AD−, BD+, and BC−). The experiments revealed that configural learning tasks are affected by hippocampal lesions with some stimulus combinations, but not others. To identify the role played by the hippocampus in solving configural discriminations we, therefore, examined how lesions in this region affect: discriminations between different media, discriminations based on distal spatial cues, and biconditional discriminations based on local or distal cues. By mapping the inter-relations between these forms of learning, the study sought to understand when configural learning depends on the hippocampus and how this ability relates to spatial learning.

## GENERAL MATERIALS AND METHODS

Through a series of planned comparisons (Table 1) the present study contrasted learning based on spatial or nonspatial cues, proximal or distal cues, and elemental versus configural (non-elemental) cues. Three cohorts of rats were tested to avoid unwanted transfer effects across experiments. The rationale was to test the specificity of the spatial biconditional deficit and determine how this relates to different forms of spatial and nonspatial learning. The spatial tasks should make few, if any, navigational demands as the choice stimuli were always visible and could be directly approached (except for Experiment 1, T-maze alternation). Because of the numbers of experiments and the use of three separate cohorts of rats, the results for each experiment are described before progressing to the next experiment.

**Table 1:** Sequence of Experiments

Exp.	Cohort	Task type	Association	Room	Cue type	Direction
1	1	T-maze alternation	Elemental	X	Spatial/Room Cues	
2	1	Simple Discrimination	Elemental	A	Digging media	Bidirectional
3	1	Biconditional Discrimination	Configural	B	Proximal Context	Bidirectional
4	1	Biconditional Discrimination	Configural	B	Spatial/Room Cues	Bidirectional
5	1	Simple Discrimination	Elemental	C	Spatial/Room Cues	Unidirectional
6	1	Biconditional Discrimination	Configural	C	Spatial/Room Cues	Bidirectional
7	2	Simple Discrimination	Elemental	B	Spatial/Room Cues	Unidirectional
8	3	Simple Discrimination	Elemental	B	Spatial/Room Cues	Bidirectional
9	3	Simple Discrimination	Elemental	D	Spatial/Room Cues	Unidirectional
10	3	Biconditional Discrimination	Configural	D	Spatial/Room Cues	Unidirectional

The Association column refers to whether the learning task required a configural solution or whether it could be solved with an elemental solution. The Direction column refers to whether the rats reached the cups from two directions (bidirectional) or from one fixed direction (unidirectional) in each test box.

### Animals

In this study, three different cohorts of rats received bilateral hippocampal lesions (Hpc1, Hpc2, and Hpc3), along with their respective control groups (Sham1, Sham2, and Sham3). Cohort 1, Cohort 2, and Cohort 3 comprised, respectively, 21, 20, and 25 male, Lister Hooded rats. All rats were housed in pairs under diurnal conditions (12 h light/12 h dark), and water was provided ad libitum throughout the study. Animals were always testing during the day, that is, during the light phase. Animals were food-deprived up to 85% of their free-feeding body weight and maintained above this level during behavioral testing. Rats were 10 months (Cohort 1), 11 months (Cohort 2), and 6 months (Cohort 3) old at the start of the study, having previously been trained on geometric discriminations in a curtained water tank that involved the rats distinguishing plain walls of differing lengths. All experiments were performed in accordance with the UK Animals (Scientific Procedures) Act, 1986 and associated guidelines.

### Surgery

Rats in each cohort received bilateral hippocampal lesions (Hpc1 = 12, Hpc2 = 10, and Hpc3 = 13) made by injecting ibotenic acid. Rats were first anesthetized using an isoflurane-oxygen mix. The rat was then placed in a stereotaxic frame (Kopf Instruments, Tujunga, CA), with the incisor bar set at −3.3 mm, and the rat administered with 0.1 mg/kg of the analgesic Metacam subcutaneously. A sagittal incision was made in the scalp, and the skin retracted to expose the skull. A dorsal craniotomy was made directly above the target region and the dura cut to expose the cortex. The rats in groups Hpc1, Hpc2, and Hpc3 received injections of ibotenic acid (Biosearch Technologies, San Rafael, CA) dissolved in phosphate-buffered saline (pH 7.4) to provide a solution with a concentration of 63 mM. The injections were made through a 2-µL Hamilton syringe held with a microinjector (Kopf Instruments, Model 5000). Fourteen infusions per hemisphere were made at an infusion rate of 0.10 µL/min and a diffusion time of 2 min [for coordinates and volume, see Iordanova et al. (2009)]. The control groups (Sham1 = 9, Sham2 = 10, and Sham3 = 12) received identical treatments except that the dura was repeatedly perforated with a 25-gauge Microlance3 needle (Becton Dickinson, Drogheda, Ireland) and no solution was infused into the brain. All rats were 3–4 months old at the time of surgery.

### Histological Procedure

On completion of behavioral testing, all rats received a lethal overdose of sodium pentobarbital (60 mg/kg, Euthatal, Rhone Merieux). The rats from Cohort 1 and Cohort 2 were transcardially perfused, first with 0.9% saline and then with 10.0% formal-saline. Their brains were extracted, post-fixed for 24 h, and then transferred to 25% distilled water sucrose solution in which they remained for a further 24 h. The rats from Cohort 3 were transcardially perfused with 0.1 M phosphate buffer saline (PBS) followed by 4% paraformaldehyde in 0.1 M PBS, so that their tissue could be used for immunohistochemistry. All brain sections were cut at 40 µm on a freezing microtome in the coronal plane. The sections were collected on gelatine-coated slides, left to dry in room temperature over 24 h, and then stained with cresyl violet, a Nissl stain.

The amount of damage in the hippocampus (dentate gyrus and CA fields but not including the subiculum) was measured separately with the program AnalysisD (Soft-Imaging Systems, Olympus). First, the total area of the region of interest was measured from ten coronal sections corresponding to −2.12, −2.80, −3.30, −3.80, −4.30, −4.80, −5.30, −5.80, −6.30, and −6.80 relative to bregma (Paxinos and Watson, 2005) in a surgical control. Then, using the same protocol, the extent of hippocampal damage and cortical damage was quantified for each animal that received a hippocampal lesion.

### Histological Analyses

### Cohort 1

**FIGURE 1:**
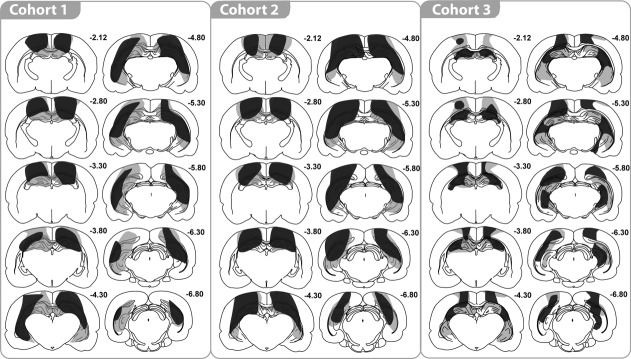
Coronal sections illustrating those cases with the smallest (dark gray) and largest (light gray) bilateral hippocampal lesions in each of the three cohorts of rats. The numbers refer to the approximate distance of each section in mm caudal to bregma.

Figure 1 depicts a series of coronal sections (adapted from Paxinos and Watson, 2005) showing the maximum and minimum extent of hippocampal damage. Histological analyses revealed that three rats had appreciable sparing of the hippocampus (<50% volume loss). These three rats were excluded from the behavioural analyses. The remaining nine Hpc1 rats had extensive damage to the hippocampus that was typically more complete in the dorsal rather than the ventral hippocampus. Three rats had >85% volume loss of the dorsal hippocampus, with three more rats having over 70% loss of the dorsal hippocampus. In these rats, the only consistent sparing was restricted to the most medial part of the dorsal hippocampus, in particular the most medial part of the blade of the dentate gyrus. The remaining three rats had from 50 to 70% cell loss in the dorsal hippocampus, with most sparing of the medial parts of the dorsal hippocampus.

In the ventral hippocampus, three rats had over 70% tissue loss, four rats had between 55 and 70% damage, and two rats had between 35 and 55% damage. Within the ventral hippocampus, all rats showed some partial sparing of the cell layers of the lateral blade of the dentate gyrus, as well as the most ventral part of CA1 and CA3.

The hippocampal lesions were confined such that no damage was visible to more ventral structures, leaving the thalamus intact. All rats did, however, sustain cortical damage dorsal to the hippocampus that invaded parts of the primary somatosensory area and adjacent cortex within the parietal area (Fig. 1). At posterior levels, parts of the primary and rostrolateral visual areas were sometimes damaged. Very limited damage to the dysgranular retrosplenial cortex was seen in all rats.

### Cohort 2

Histological analyses (Fig. 1) revealed that one rat had considerable sparing of the hippocampus (<50% volume loss) and was excluded from the behavioral analyses. The remaining nine Hpc2 rats had greater cell loss in the dorsal than the ventral parts. In five cases over 70% of the dorsal hippocampus was lost, and cell sparing was confined to the medial most parts of the hippocampus, including the medial limit of the dentate gyrus blade. The remaining four Hpc2 rats, which had between 60% - 70% volume loss of the dorsal hippocampus, again showed more sparing of the medial part of the dorsal hippocampus, including the medial dentate gyrus.

There was appreciable cell loss in the ventral hippocampus, although all rats showed some sparing in the lateral blade of the dentate gyrus, as well as the most ventral parts of CA1 and CA3. In three rats, there was over 70% damage to the ventral hippocampus. One rat had 58% damage to the ventral hippocampus while the remaining five rats had between 30% - 50% volume loss. Again, the lesions did not extend into the thalamus, although in all cases there was cortical damage dorsal to the hippocampus. This cortical damage included parts of the primary somatosensory area and the parietal region of the posterior association area. At more posterior levels, there was often restricted damage to parts of the primary and rostrolateral visual areas. The cortical damage often extended medially to reach the lateral edge of the dysgranular retrosplenial cortex.

### Cohort 3

Four animals were excluded from further analysis. In two of these cases, there was excessive hippocampal sparing (less than 40% damage). A further animal was excluded because of widespread cortical damage, while the lesion in the fourth case was largely unilateral. In the remaining nine Hpc3 cases, the volume loss for the entire hippocampus was between 42% - 79% (Fig. 1). As before, the cell loss was greater in the dorsal hippocampus where six cases had more than 70% damage. In the remaining three cases, the dorsal hippocampus sparing (range: 48% - 53%) extended into lateral CA3, and sometimes into the medial portion of CA1. The only subfield to show any consistent partial sparing was the dentate gyrus, but here the subfield was markedly diminished in volume despite spared granule cells. The dorsal subiculum was damaged in all cases, often being extensively damaged.

Tissue loss in the ventral hippocampus ranged from 27% - 69%, with any sparing in the most ventral part of the CA1 and CA3, as well as in the dentate gyrus. The ventral subiculum was typically spared. It should be added that in all nine cases the hippocampus was markedly shrunken in all three planes, and so it is likely that the coronal reconstructions underestimated the extent of tissue loss. In eight cases the lesions just encroached into the dorsal thalamus. Five of these cases had partial damage to the laterodorsal nucleus, which in one case included unilateral damage to the most dorsal part of the anterior ventral thalamus. Finally, all rats also had some cell loss in cortex dorsal to the hippocampus. As before, the damage involved parts of the primary and secondary motor areas, the primary somatosensory area, and the parietal region of the posterior association area. There was also some restricted damage in dysgranular retrosplenial cortex.

### Statistical Analyses

Performances in the T-maze alternation, digging media discrimination (Experiment 2), proximal context biconditional task (Experiment 3), and distal spatial biconditional tasks (Experiments 4, 6, 10) were analyzed using a one between-subject (groups) by one within-subject (sessions) ANOVA. For the various go/no-go discriminations (Experiments 5, 7, 8, 9) a three-way ANOVA was used to analyze the performance of the animals: one between factor (groups) and two within-subjects (sessions and latency on go/no-go trials). When there was an interaction, simple effects were examined (Howell, 1982). The ratio of the latencies for the ‘no-go’ and the ‘go’ trials was also considered to help clarify any experiments with seemingly borderline lesion effects.

### Behavioral Findings

Aside from T-maze alternation, the findings from all the following experiments are summarized in Table 2. This table highlights the contrasting effects of the hippocampal lesions on the various elemental and biconditional discriminations.

As explained, for the clarity of the paper, the Materials, Methods and Results are presented for each experiment before going to the next experiment.

**Table 2:** Summary of Behavioral Findings

Task	Direction	Experiment	Cohort	Impact of hippocampal lesions
Spatial alternation T-maze	N.A	1	1	**X**
Simultaneous nonspatial discrimination	Bidirectional	2	1	√
Spatial go/no-go discrimination	Unidirectional	5, 7, 9	1, 2, 3	√
	Bidirectional	8	3	**X**
Biconditional discrimination with proximal context cues	Bidirectional	3	1	√
Biconditional discrimination with distal spatial cues	Bidirectional	4, 6	1	**X**
	Unidirectional	10	3	**X**

√:No hippocampal lesion deficit; **X**: Hippocampal lesion deficit.

The direction terms refer to whether the rats reached the cups from two directions (bidirectional) or from one fixed direction (unidirectional) in each test box.

## COHORT 1

## EXPERIMENT 1: SPATIAL ALTERNATION IN THE T-MAZE

In order to verify the efficacy of the lesions, before commencing the proposed studies, the Hpc1 and Sham1 rats were first trained on reinforced alternation in the T-maze. This task is highly sensitive to hippocampal damage (Aggleton et al., 1986; Bannerman et al., 1999). Cohorts 2 and 3 were not tested on T-maze alternation as they had identical surgical protocols to Cohort 1.

### Materials and Methods

#### Apparatus

Each arm of the T-maze was 70 cm long and 10 cm wide, and made of wood. The walls were 17 cm high and made of clear Perspex. A moveable aluminum barrier was used to block access to a particular T-maze arm in the Sample Phase, and was then re-positioned 25 cm from the base of the start arm to create a start area for the Test Phase. The maze was supported by two stands (94 cm high) and situated in a rectangular room (280 x 300 x 240 cm) with salient visual cues (posters and high contrast images) on the walls. The illumination level in the centre of the room was 560 lux.

#### Procedure

Following two pre-training days, during which the rats were trained to eat reward pellets (45 mg sucrose pellet; Noyes Purified Rodent Diet; UK) at the two opposite ends of the arm of the T-maze, the test proper began. Each trial comprised two stages. In the Sample Phase, the rat was released from the start area (at the base of the T-maze) and by placing an aluminum barrier at the choice point was forced to enter the one open arm. The rat was then allowed to consume one sucrose pellet at the end of the arm. The animal was then picked up and confined in the start box for a delay of 10 sec, during which time the aluminum block was removed. At the start of the Test Phase the door to the start arm was opened and the animal allowed a free choice between the two arms of the T-maze. The rat was rewarded with a single pellet for visiting the arm not entered on the Sample Phase. Rats received spaced trials so that the inter-trial interval was typically four minutes, and there were six trials in a session. All rats received six sessions (one session per day).

#### Results

As expected, the Hpc1 rats were severely impaired at spatial alternation in the T-maze (*F*_(1,16)_ = 109.7, *p* < 0.001). There was a significant effect of session (*F*_(5,80)_ = 2.93, *p* < 0.05), reflecting modest improved learning of the task by the Sham1 rats. The overall percent correct alternation scores for Sham1 were 79.0% (SEM ±0.10) but only 50.4% for the Hpc1 rats (SEM ±0.14). The Hpc1 scores did not differ from chance (50%). There was no group by session interaction (*F*_(5,80)_ = 1.01, *p* = 0.42) with the groups differing from the very first session (the Sham1 rats performed at over 70% on the first session).

## EXPERIMENT 2: SIMULTANEOUS NONSPATIAL DISCRIMINATION (MEDIUM A VERSUS MEDIUM B)

This experiment tested whether hippocampal lesions impair an elemental discrimination in which digging in only one of two different media was rewarded.

### Materials and Methods

#### Apparatus and room

**FIGURE 2:**
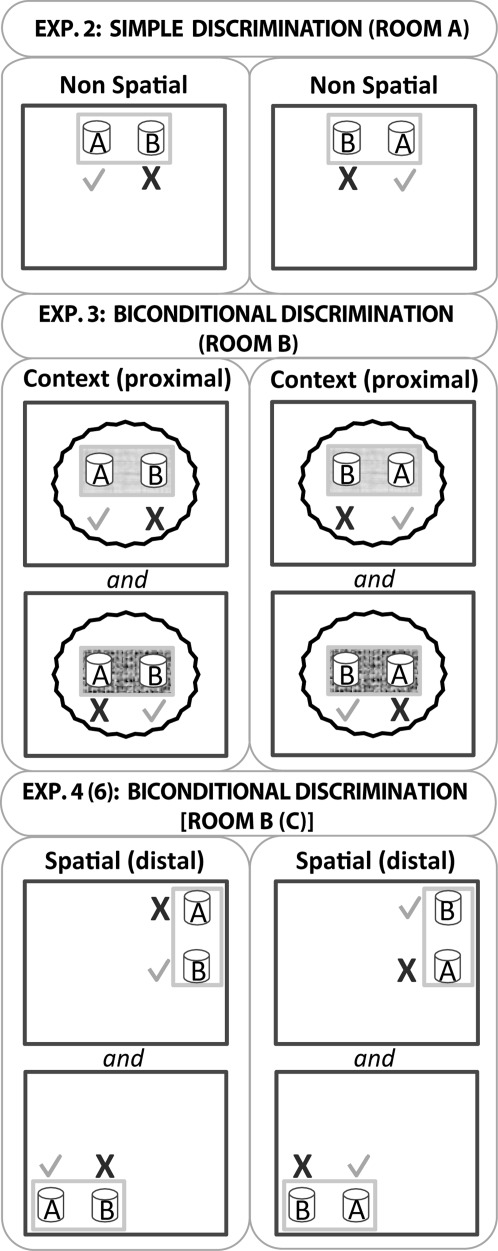
Schematic representation of the testing arrangements for Experiments 2–4. The upper figure depicts the simple nonspatial discrimination (digging medium A versus digging medium B, Experiment 2). One of these media always contained a food reward (tick), irrespective of position, while the other did not (cross). The light gray rectangular outline represents the test box while the darker gray outline represents the room. Note, the test box is not drawn to scale with respect to the room. The middle figure depicts the first biconditional task (Experiment 3), in which the proximal context within the test box determined whether medium A or B contained food. In the version portrayed, digging in A not B was rewarded in the paler context, irrespective of the left/right location of medium A. The converse contingency applied to medium B. The wavy line represents the curtain that occluded distal room cues. The lower figure represents the spatial biconditional discriminations used in Experiment 4 (room B) and Experiment 6 (room C). Medium B is correct in the northeast corner of the room irrespective of where the digging cup is placed within the text box (gray rectangle). Conversely, medium A is correct in the south west corner. All other conventions as above. Note that Room C used for Experiment 6 contained a water maze, but all testing was outside this maze.

Animals were tested in a white plastic test box (40 cm long x 20 cm wide x 12.5 cm high). Each digging cup was placed in the middle of the opposing short walls of the rectangular test box, 22 cm apart (see Fig. 2 upper). The digging cups consisted of a black plastic cylinder with an internal diameter of 7 cm and a height of 6 cm. A gray plastic square (9 cm x 9 cm) was fixed to the base of each cylinder. Velcro secured the cups to the box floor, so rats could not upset the cups. During pretraining the two cups were identical (both plain black), but thereafter the cup containing one medium had checkered tape attached to the outside while the cup containing the other medium remained plain black. The digging media consisted of either small multi-colored beads or shredded red paper. The food reward was half a loop of a single Cheerio (Nestle, UK) that was buried in the digging media. To discourage rats from trying to locate the food reward by its scent, a perforated metal grid was placed inside the cup to create a false bottom. Cereal loops were placed under this grid, where they could not be retrieved by the rats and they were replaced with fresh cereals twice a week. In addition, cereal crumbs were mixed with the digging medium to ensure that both the correct and incorrect choices smelt of the food reward. Both pre-training and testing for the simple discrimination took place in the same room (Room A). Room A was relatively narrow (330 cm long x 190 cm wide x 256 cm high) compared to the other rooms used in this study. Visual cues, such as posters and shelves were fixed on the walls. A table was placed near the back wall of the room. The illumination level where testing took place was 430 lux.

#### Pretraining

Rats were placed singly in the white plastic test box with two identical digging cups filled with sawdust. First a reward was placed on top of the medium. Then, the reward was buried increasingly deep so that rats had to dig to find the food. Every time the rat found the food the cup was re-baited, and so on for 10 min. Both cups were baited. Pre-training lasted between four and six days, when all rats were reliably digging to retrieve the rewards.

#### Procedure

Three or four rats were simultaneously brought to the test room in an enclosed carrying box made of aluminum. Each rat was in a separate container and could not see the surrounding environment. Two digging cups, one filled with multicolored beads and the second filled with red shredded paper, were placed at opposite ends of the rectangular test box (40 cm apart). As shown in Figure 2 (upper), only one medium was associated with a reward, so that for half of the rats multi-colored beads were correct and for the other half, red shredded paper was correct. On each trial, the rat was set down in the middle of the white test box, midway between the two cups (one to the right, the other to the left), and allowed to explore the cups. Selecting a cup corresponded to when the rat first moved the medium with its paw or snout. When the rat dug in the baited cup, the animal could retrieve the reward and a correct choice was scored, but when the rat dug in the unbaited cup, an incorrect choice was scored and the correct cup was removed. On such trials, the rat was left in the apparatus for an extra 5 s before being taking out of the box and placed back in the enclosed carrying box. Rats were run in spaced trials, i.e., four rats were run one after the other for trial 1, then the four animals for trial 2, and so on. Consequently there was an inter-trial interval of ∼2-3 min. The criterion for training was set at 80% for the Sham1 group mean. Training involved two sessions, one per day, each containing 16 spaced trials. The position of the correct cup (left or right from the rat's start position) changed randomly across trials.

### Results

**FIGURE 3:**
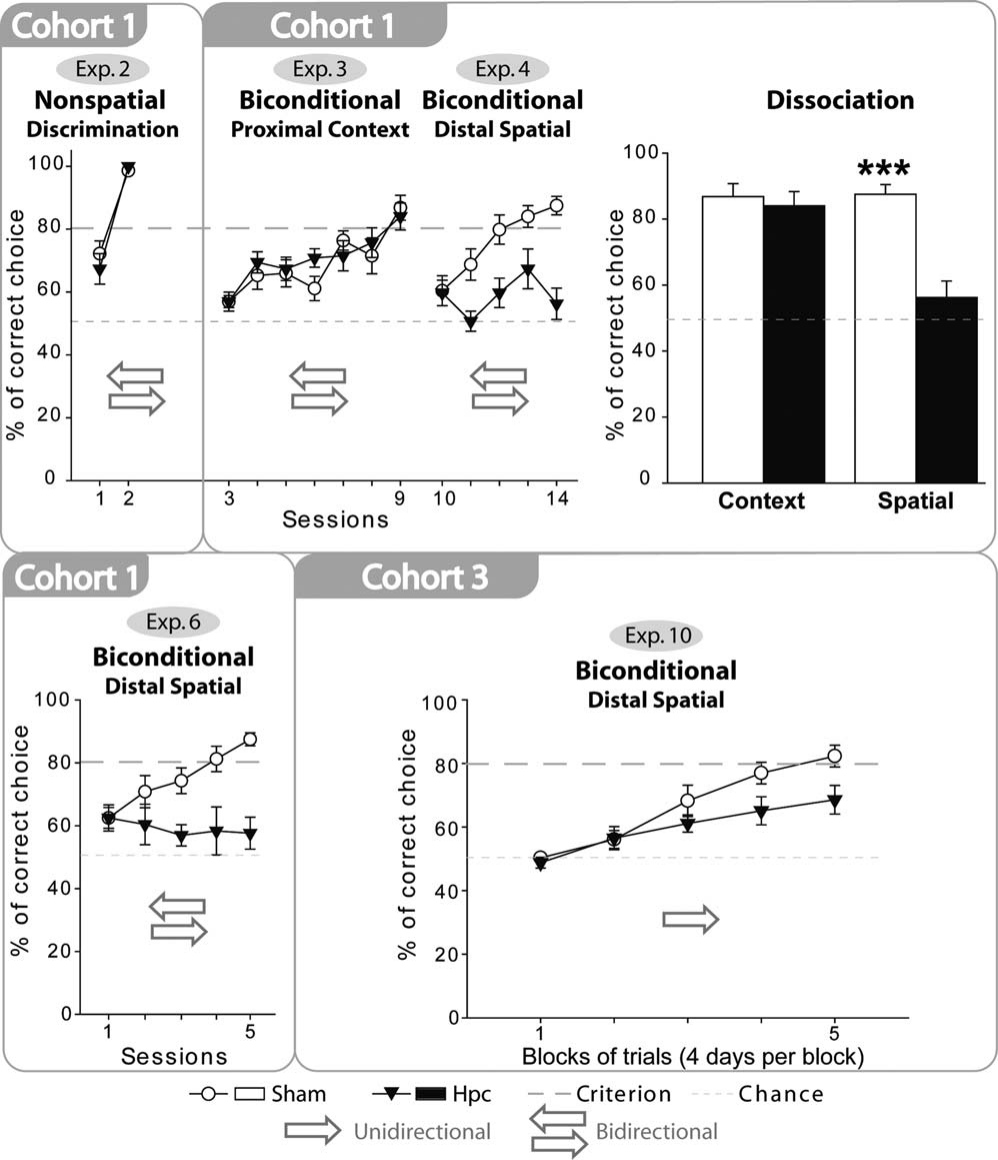
Series of graphs that show the mean performances (+/− standard error) of the rats with hippocampal lesions (black triangles) and their controls (white circles) on a simple discrimination (Experiment 2, medium A versus medium B) and on a series of biconditional discriminations. In all experiments, chance was 50% while the criterion was set at 80%. The arrows show whether the rats reached the digging cups from two directions or from one fixed direction in each test box. The bar chart depicts the scores of the two groups on the final session of the context (appearance of test box) biconditional task and the spatial (room location) biconditional task, highlighting the very different impact of hippocampal lesions on performance. *** *P* < 0.001.

Both the rats with hippocampal lesions (Hpc1) and their controls learnt to choose the correct digging medium, and the two groups did not differ in their overall performance (Fig. 3; lesion effect *F* < 1). By the second session both groups performed above the 80% criterion (session effect *F*_(1,16)_ = 85.5, *p* < 0.001). There was no evidence of lesion by session interaction (*F* < 1). Consequently, the hippocampal lesions did not appear to affect the ability to discriminate the digging media.

## EXPERIMENT 3: BICONDITIONAL DISCRIMINATION WITH PROXIMAL CONTEXT CUES

This experiment examined whether hippocampal lesions disrupt the acquisition of a configural task in which local cues (proximal) within the test box indicated whether to dig in medium A or medium B when they were placed side-by-side (Table 1). More distal cues were occluded by a circular curtain that surrounded the test box.

### Materials and Methods

#### Apparatus and room

For this task, digging cups were placed in one of two different plastic boxes (both 33 x 26 x 16.5 cm). The two boxes could readily be distinguished as one box had laminated wall panels composed of white and red triangles, and also had a green, textured Duplo (Lego, UK) base covering the floor. The second box had a smooth, checkered (black and white) laminated floor, but the walls were plain.

Training took place in a new room from Experiment 2 (Room B). Room B was square (280 cm long x 280 cm wide x 256 cm high). An opaque curtain fixed to a circular panel on the ceiling was drawn around the test boxes to block distal cues. The illumination level in the text boxes was 452 lux. The two digging pots were 22cm apart in each box.

#### Procedure

Animals were trained on a biconditional task, in which a specific digging medium was only correct in a specific context (see Fig. 2 mid). The rats had, therefore, to learn two concurrent rules. Rule 1: multi-colored beads were correct when presented in the checkered floor box (context 1). Rule 2: red shredded paper was correct when presented in the Duplo base box (context 2). On each trial, a rat was placed midway between the two cups (one to the right, the other to the left) and allowed to choose. Animals received 16 trials per session (8 trials of Rule 1 and 8 trials of Rule 2), with trial types in a randomized sequence. The rats were run in spaced trials, i.e., the groups of three or four rats were run one immediately after the other for every trial. Trial types and the location of the correct cup inside the box (left or right) were counterbalanced within sessions and across groups. The test boxes were placed on a table in the center of room B, and so were always in the same location. Only the local context changed between trials. The Sham1 group performance criterion was set at 80% (for one session), before stopping the experiment. All rats received seven sessions, one per day.

### Results

Both the rats with hippocampal lesions (Hpc1) and their controls (Sham1) readily learnt the contextual biconditional task (Fig. 3), and there was no significant group difference (*F* < 1). Both groups required seven sessions to reach the 80% criterion (the session effect reflecting improved behavioral performance *F*_(6,96)_ = 13.7, *p* < 0.001) across the two contexts. No lesion by session interaction was found (*F*_(6,96)_ = 1.04, *p* = 0.41). Consequently, the hippocampal lesions did not appear to affect the ability to learn a biconditional discrimination that required the use of floor and wall cues immediately adjacent to the digging cups to guide the choice of the correct digging medium.

## EXPERIMENT 4: BICONDITIONAL DISCRIMINATION WITH DISTAL SPATIAL CUES (ROOM B, BIDIRECTIONAL TRIALS)

This experiment examined whether hippocampal lesions disrupt the acquisition of a configural task in which distal spatial cues beyond the test box indicated whether to dig in medium A or medium B to find food (Table 1). Local cues (proximal) were held constant across the conditions.

### Materials and Methods

#### Apparatus and room

The single test box in which the digging cups were presented was identical to the box used in Experiment 2 (white plastic). Training took place in Room B (same room as Experiment 3), with the test box set on tables placed in the two diagonally opposite corners, 180 cm apart. The room was free of obstacles so that all walls were visible from any corner of the room. Posters and shelves were fixed to the walls. The room was illuminated with eight spot bulb lights fixed to the ceiling. The illumination levels in the two corner locations were 108 and 151 lux, respectively.

#### Procedure

Using procedures identical to those in Experiment 3, the Hpc1 and Sham1 rats were trained on a new biconditional task. Now, the task was to learn which medium was correct in which location. Consequently, the new biconditional rule was that multicolored beads (but not shredded paper) were correct in one corner of the room (location 1), while shredded paper (but not multicolored beads) was correct in the diagonally opposite corner of the room (location 2; Fig. 2 lower). The single test box was moved between the two locations between trials and its orientation varied in the manner shown in Figure 2 according to the corner in which it was located. The test box was always 20 cm away from the walls. There was no curtain surrounding the test boxes (unlike Experiment 3), so animals could use distal spatial cues to solve the task. Once again, rats were set down in the middle of the test box at the start of a trial, that is, placed midway between the two cups (one to the right, the other to the left). Rats were trained for five days with 16 trials per day. The training criterion was set at 80% for the Sham1 group (mean performance over one session). This criterion was reached after three days. However, to verify whether the hippocampal animals could learn this critical experiment, training was carried on for two extra days.

### Results

The spatial biconditional task consisted of choosing the correct medium in the correct location (e.g., one of the corners of the room; Fig. 2). Now the Sham1 rats outperformed the Hpc1 rats (*F*_(1,16)_ = 14.4, *P* < 0.01; Fig. 3). In addition to an overall improvement in performance (session effect, *F*_(4,64)_ = 8.39, *P* < 0.001) there was a lesion by session interaction (*F*_(4,64)_ = 4.85, *P* < 0.01) reflecting the hippocampal lesion deficit. Subsequent analyses (simple effects) revealed that the control rats outperformed the Hpc1 rats in Sessions 2–5 (Session 2: *F*_(1,80)_ = 8.02, *P* < 0.01; Session 3: *F*_(1,80)_ = 9.98, *P* < 0.01; Session 4: *F*_(1,80)_ = 6.83, *P* < 0.05; Session 5: *F*_(1,80)_ = 24.0, *P* < 0.001), but not in Session 1 (*F* < 1). By the end of this experiment (Session 5), Sham1 rats were above the 80% criterion in each location, whereas the Hpc1 rats remained at chance (one sample *t*-test: *t*_(8)_ = 1.25, *P* = 0.25). Consequently, the hippocampal lesions severely disrupted the ability to acquire a spatial biconditional task in which distal room cues guided the choice of the correct digging medium.

Direct comparisons between the local context biconditional (Experiment 3) and distal spatial biconditional (Experiment 4) could be made using data from the final session of both experiments (Fig. 3). Of particular interest was the lesion by experiment interaction (*F*_(1,16)_ = 15.7, *P* < 0.001), reflecting the selective poorer performance of the Hpc1 rats in the spatial biconditional learning task. Further analyses of the simple effects confirmed that the groups did not differ in the proximal context task (*F* < 1), but that the rats with hippocampal lesions were severely impaired on the spatial biconditional task (*F*_(1,32)_ = 28.8, *P* < 0.001).

## EXPERIMENT 5: SPATIAL GO/NO-GO DISCRIMINATION (ROOM C, UNIDIRECTIONAL TRIALS)

**FIGURE 4:**
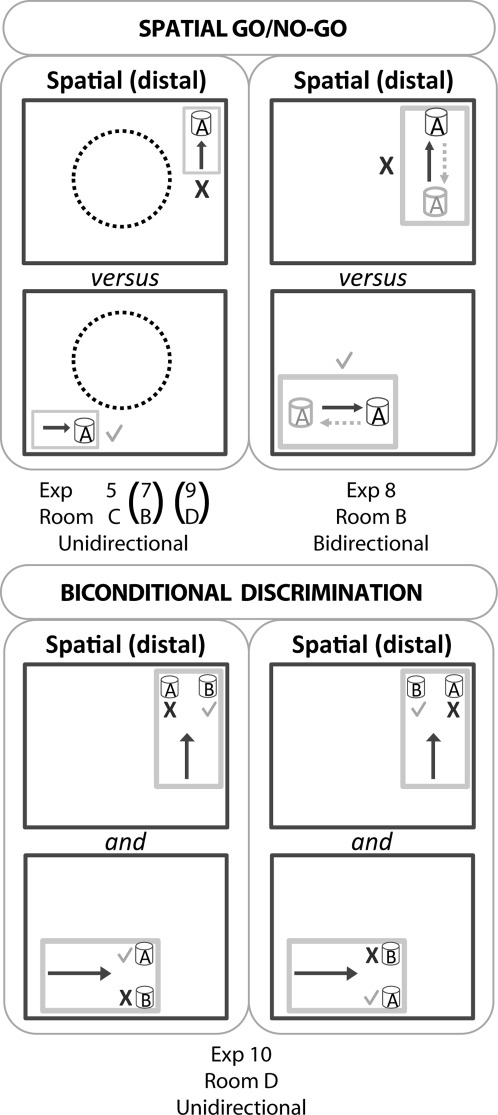
Schematic representation of the testing arrangements for Experiments 5, 7, 8, 9, and 10. The upper figure depicts the four go/no-go discriminations. In these discriminations, digging when the test box was in one location was rewarded but digging when the text box was in the diametrically opposite location was unrewarded. Consequently, a tick corresponds to a correct choice, whereas a cross corresponds to an incorrect choice. The light gray rectangular outline represents the test box while the darker gray outline represents the room. The arrows indicate the direction of travel to reach the digging cup. Note: the test box is not drawn to scale with respect to the room. Upper left: In three experiments (5, 7, 9) rats only approached the digging cup from one direction in each test box, as shown by the arrow. In Experiment 5, the center of test room (C) contained a circular water maze (dashed circle). Room B (Experiment 7) and Room D (Experiment 9) were, however, clear, that is, the full room could be viewed from each corner. The lower figure depicts the spatial biconditional task used in Experiment 10. In this task, medium B was correct when in the northeast corner, irrespective of whether it was placed to the right or left. Conversely, medium A was always rewarded in the southwest corner. The task, which took place in Room D and followed immediately after Experiment 9, was designed so that the rats could approach the choice stimuli in a constant direction for a given location. For this reason, it was necessary to use a larger test box (which was also used in Experiment 9). Again, the test box is not drawn to scale.

The purpose of this experiment was to test why the Hpc1 rats were impaired on the biconditional discrimination that involved distal spatial cues (Experiment 4) yet unimpaired on the biconditional that relied on proximal context cues (Experiment 3). One possibility was that the Hpc1 rats could not effectively discriminate the distal spatial cues. This possibility was tested in a go/no-go task involving the discrimination of two different room locations. A new room (Room C) was used to minimize transfer effects from the previous experiments. In the middle of this room was a circular water maze (2 m diameter), and testing took place in two opposite corners of the room, that is, outside the water maze (Fig. 4 upper left). When an animal was tested in one location, it could not see the opposite location.

### Materials and methods

#### Apparatus and room

Room C measured 360 cm long, 300 cm wide, and 244 cm high. A raised water-maze (2 m diameter) was fixed to the centre of the room, and because of its position and height, the animals were unable to see the opposite corner of the room. The light source was four daylight fluorescent tubes fixed on the walls (not on the ceiling). The single test box was identical to that used in Experiments 2 and 4. The light levels in the two corners were, respectively, 108 and 237 lux. The test box was always 20 cm away from the walls. Only a single digging cup was used throughout (the same plain black cup described in Experiment 2).

#### Procedure

On each trial a single cup was filled with sawdust and placed at the mid length of one of the short walls of the white plastic test box. The white box could be placed in one of two locations in diagonally opposite corners of room C. Thus, for any given rat, the cup was always baited in one room location (go response), but never baited when located in the other room location (no-go response; Fig. 4 upper left). The test box was always placed on one of two tables that were situated in the two diagonally opposite corners of the room (450 cm apart). Learning was assessed by comparing the latency of the rat to dig when the box was in the baited location and the latency to dig when the box was in the never-baited position, where the rat should learn to withhold from digging. Each trial had a time limit of 20 s, after which the rat was removed. If the rat dug in the medium before 20 s in the correct location, the rat was removed as soon as it had consumed the cereal reward; but if the rat dug in the incorrect location it was left for an extra 5 s before being removed from the box. The trial order was counterbalanced between the two locations (correct and incorrect). Because the rats were always released from the same end of the test box, all trials in each location were in a particular direction (“unidirectional”), with the direction differing between the two test locations (Fig. 4). It was deliberately decided that the two locations should involve different principal directions of travel (Fig. 4) as this arrangement ensured that for all trials the rats ran with a wall closer to their right flank, irrespective of location. The concern was that the relative position of the rat to the nearest wall would provide a highly salient cue (see also Experiments 7,9). Animals were trained for four days.

### Results

**FIGURE 5:**
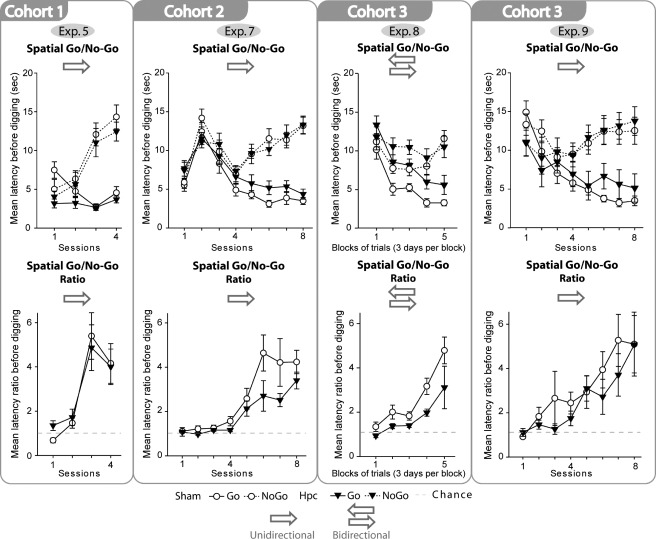
Performances of the rats on four spatial discrimination tasks (go/no-go). The graphs show the mean group scores (+/− standard error) of the rats with hippocampal lesions (black triangles) and their controls (white circles). The four upper graphs compare the latencies to dig on both rewarded trials (“go,” solid lines) and nonrewarded trials (“no-go,” dotted lines). With task acquisition, these graphs diverge. The four lower graphs show the same data expressed as means of the individual ratios (latency to dig on a “no-go” trial divided by latency to dig on a “go” trial). With task acquisition the ratio scores rise. The arrows show whether the rats reached the cups from two directions (bidirectional) or from one fixed direction (unidirectional) in each test box.

A food reward cup was baited in one location in Room C, but not baited in a second location (Fig. 4). There was no evidence that the Hpc1 rats were impaired as both groups learnt to withhold digging in the never-baited location (Fig. 5). Consequently, there was a significant discrimination effect (*F*_(1,16)_ = 63.7, *P* < 0.001), that is, the latency to dig was higher in the incorrect location (no-go) but there was no overall latency difference between the two groups (*F*_(1,16)_ = 2.42, *P* = 0.14; see Fig. 5). The lack of any lesion effect is supported by the absence of a lesion by discrimination interaction (*F* < 1), while the performance by session interaction (*F*_(3,48)_ = 33.8, *P* < 0.001) reflects the ability of the rats to differentiate the two locations with training (i.e., dig in the correct location, but withhold their response in the incorrect location). The ratios between the no-go and go trials were also calculated (Fig. 5, lower), but again there was no evidence of a lesion effect (*F* < 1). The conclusion was that rats with hippocampal lesions could effectively solve a place discrimination task (when each location was approached from a constant direction).

## EXPERIMENT 6: BICONDITIONAL DISCRIMINATION WITH DISTAL SPATIAL CUES (ROOM C, BIDIRECTIONAL TRIALS)

### Materials and Methods

The intact performance of the Hpc1 rats in Experiment 5 (spatial go/no-go discrimination) contrasted with the spatial biconditional learning deficit in Experiment 4. Experiments 4 and 5 were, however, carried out in different rooms to reduce transfer effects. To determine whether having learnt a spatial go/no-go discrimination (Experiment 5) the Hpc rats could use that same spatial information to solve a biconditional discrimination, Experiment 6 used the same locations in Room C for a biconditional discrimination (Fig. 2 lower). The apparatus and training procedure were identical to the biconditional task in Experiment 4, for example, rats were always released from the middle of the plastic test box, and only the test room differed (see Fig. 2 lower). Rats were trained for five days and the group criterion was again set to 80%.

### Results

In keeping with Experiment 4, the Hpc1 rats were severely impaired compared to the Sham1 rats (*F*_(1,16)_ = 12.0, *P* < 0.01; Fig. 3). The lesion by session interaction reflected the superior performance of the Sham1 rats, which emerged with training (*F*_(4,64)_ = 4.39, *P* < 0.01). Subsequent analyses of the simple effects revealed that the two groups did not differ during Session 1 (*F* < 1) and Session 2 (*F*_(1,80)_ = 2.37, *P* = 0.13) but, thereafter, the Hpc1 group was impaired (Session 3: *F*_(1,80)_ = 6.59, *P* < 0.01; Session 4: *F*_(1,80)_ = 11.58, *P* < 0.001; Session 5: *F*_(1,80)_ = 19.5, *P* < 0.001). By the end of Session 5, the Sham1 rats were above the 80% criterion in each location, while the scores of the hippocampal rats, as in Experiment 4, did not differ from chance (Hpc1 group, one sample *t*-test: *t*_(8)_ = 1.50, *P* = 0.17). The conclusion was that rats with hippocampal lesions could not solve a spatial biconditional task even though spatial cues in the same room could be effectively discriminated (when each location was approached from a constant direction).

## COHORT 2

## EXPERIMENT 7: SPATIAL GO/NO-GO DISCRIMINATION (ROOM B, UNIDIRECTIONAL TRIALS)

### Materials and Methods

This experiment had two goals: (1) to test the reliability of the spared location learning in Experiment 5, and (2) to use the same location cues as in Experiment 4, where hippocampal lesions produced a biconditional learning deficit. The procedure was identical to Experiment 5 (Fig. 4 upper) except for the use of Room B, not Room C. A new cohort (Cohort 2) was tested in order to minimize transfer effects. The illumination levels in the two test locations were matched at 123 lux. Rats were trained for 8 days.

### Results

To test whether the spared performance on the go/no-go spatial discrimination found in Experiment 5 (Room C) was reliable, the second cohort of rats was tested on a spatial discrimination in Room B. The locations matched those used in Experiment 4, where the Hpc1 rats were impaired on a spatial biconditional task. From Figure 5, it is evident that both groups could now discriminate the two locations. For this reason, there was a significant discrimination effect (Fig. 5), that is, shorter latencies to dig in the correct location (*F*_(1,17)_ = 136.8, *P* < 0.001), as well as an improvement with training (session effect, *F*_(7,119)_ = 15.9, *P* < 0.001). Although the lesion by discrimination interaction was nearly significant (*F*_(1,17)_ = 3.81, *P* = 0.068) there was no overall effect of surgery on the total latencies (*F* < 1). The significant discrimination performance by session interaction reflected task acquisition (*F*_(7,119)_ = 27.4, *P* < 0.001).

In view of the borderline lesion by discrimination interaction, the data were reanalyzed as ratio scores (see Fig. 5). Again, there was a borderline lesion effect that narrowly failed to reach significance (*F*_(1,17)_ = 4.20, *P* = 0.056). There was a session effect as the ratios increased with learning (*F*_(7,119)_ = 16.4, *P* < 0.001) but no lesion by session interaction (*F*_(7,119)_ = 1.48, *P* = 0.18). In conclusion, the Hpc2 rats could clearly differentiate the two locations despite evidence of a marginal deficit.

## COHORT 3

## EXPERIMENT 8: SPATIAL GO/NO-GO DISCRIMINATION (ROOM B, BIDIRECTIONAL TRIALS)

### Materials and Methods

This experiment examined why the Hpc1 and Hpc2 rats were impaired on the spatial biconditional task (Experiments 4 and 6) that involved room cues, but not impaired or seemingly less impaired on the spatial go/no-go discriminations (Experiments 5 and 7). One possible explanation for the relative sparing in Experiments 5 and 7 was that all trials in a given location were run in a single direction (unidirectional), so restricting cue overlap between the two locations (see Gaffan and Harrison, 1989; Dumont et al., 2007; Rudy, 2009). For this reason, rats were again trained on a go/no-go location discrimination but now the release point for each trial within a given test box alternated, that is, the rats ran in two directions at each of the two places (“bidirectional”), resulting in two digging sites within each text box. Irrespective of the direction of running, it was the location of the test box that determined whether the digging cup was baited (Fig. 4 upper right). Rats were trained for 15 days.

#### Apparatus and room

For this experiment, animals were tested in Room B (also used for Experiments 3, 4, and 7), with the same white plastic test box as for Experiments 2, 4, 5, 6 and 7.

#### Procedure

Animals were run as in Experiments 5 and 7. The only difference was that now the rats were run in one of two directions to reach the digging cup, that is, the start location was either to the West or to the East end of the box (Fig. 4 upper right). All trials were counterbalanced according to a pseudo-random schedule with the following rules: (1) the rat ran to the cup from both directions equally (i.e., 8 trials each) and (2) the rat ran toward the digging cup in the same direction for a maximum of three consecutive trials. The illumination levels at both locations were 123 lux.

### Results

A difference between the previous go/no-go tasks and biconditional discriminations was that in the latter tasks the rat could approach the digging cups in a given room location from two different directions. For this reason, the Cohort 3 rats were trained on a go/no-go discrimination in which the trials from each of the two locations were run in two opposing directions, so matching the biconditional task arrangement. Latencies to dig (Fig. 5) showed that now the Hpc3 rats were impaired compared with Sham3 rats (*F*_(1,19)_ = 5.43, *P* = 0.031). The group by condition interaction failed, however, to reach significance (*F*_(1,19)_ = 3.66, *P* = 0.071). There was a significant effect of block, condition (go/no-go), and condition by block interaction (all *P* < 0.001), all reflecting task acquisition. None of the other interactions were significant (*P* > 0.1).

Again, in view of the borderline group by condition interaction, the data were reanalyzed as ratio scores (Fig. 5). The Hpc3 group was significantly impaired (group effect *F*_(1,19)_ = 6.70, *P* = 0.018). There was a significant effect of block indicating that both groups decreased their latencies to dig in the correct location (*F*_(4,76)_ = 18.4, *P* < 0.001). The group by block interaction was not significant (*F*_(4,76)_ = 1.13, *P* = 0.35). In conclusion, although the Hpc3 rats could discriminate two locations when each was approached from two directions, performance was significantly inferior to that of the control rats.

## EXPERIMENT 9: SPATIAL GO/NO-GO DISCRIMINATION (ROOM D, UNIDIRECTIONAL TRIALS)

To provide a contrast with Experiment 8 (where rats were impaired), the rats were again trained on a spatial go/no-go task when each reward cup was approached from a single direction (“unidirectional,” see Fig. 5 upper left). Experiment 9 was also used as a first stage for the next experiment, so that the rats could first be trained to discriminate the very same spatial cues that would help solve the final biconditional task (Experiment 10).

### Materials and Methods

#### Apparatus and room

The experiment required a larger transparent test box (52 cm long × 33 cm wide × 17 cm high) than in the previous experiment so that it could be used for both this and Experiment 10 (in which two cups were placed side by side at one end of the box). For Experiment 9, a single plastic digging cup was placed midway along one of the short walls of the rectangular box. To reduce transfer effects from the previous experiments, animals were tested in a new room. Room D was 300 cm long × 277 cm wide × 241 cm high. Extra-maze cues included several operant chambers, a door, and visual cues displayed on the walls. The center of the room was empty. The room was illuminated with fluorescent strip lights. The two test locations were in diagonally opposite corners on elevated shelves, with the test box >15 cm from the side walls. The illumination levels in the two locations were 394 and 389 lux.

#### Procedure

The procedure was identical to Experiments 5 and 7 as the rats were always released from the same end of the box. The reward contingency was unchanged from these experiments, that is, digging in the single cup was rewarded in location 1 (“go response”), not rewarded in location 2 (“no-go response”). Rats were trained for 8 days, one session per day.

### Results

Now the Hpc3 rats seemed unimpaired. Based on their latencies to dig, the rats could discriminate the correct locations (trial type *F*_(1,19)_ = 32.7, *P* < 0.001). There was also a significant training effect (session, *F*_(7,133)_ = 5.48, *P* < 0.01; Greenhouse-Geisser correction for violation of sphericity) as the latencies for the incorrect location increased whereas the latencies for the correct location decreased as training progressed. There was no significant group effect (*F* < 1; see Fig. 5) and no three way interaction between trial type, group, and session (*F* < 1). Reanalyzing the data as ratios (Fig. 5) also failed to find evidence of a hippocampal deficit (group effect *F* < 1), while the effect of session (*F*_(1,33)_ = 7.41, *P* < 0.001) reflected task acquisition. There was no group by session interaction (*F* < 1). Thus, as in Experiment 5, rats with hippocampal lesions could effectively learn a spatial discrimination when the two locations were approached from a constant direction.

## EXPERIMENT 10: BICONDITIONAL DISCRIMINATION WITH DISTAL SPATIAL CUES (ROOM D, UNIDIRECTIONAL TRIALS)

In all of the previous biconditional discriminations (Experiments 3, 4, and 6), the rat was placed midway between the two digging cups, and so approached them both from different directions (bidirectional). By having two directions in each location, it is possible that task difficulty increased. The present experiment assessed biconditional learning with location cues, but now the task was simplified by having the rats only approach the two digging cups from a single direction in each location (Fig. 4 lower). For this reason, a slightly larger test box was required. Because the experiment used exactly the same locations as the previous go/no-go task (Experiment 9), it could be assumed that both groups were able to discriminate location cues that would enable the solution of the biconditional problem.

### Materials and Methods

#### Apparatus and room

Animals were tested in room D and in the same test box as Experiment 9.

#### Procedure

The rules were the same as Experiment 4, that is, choose red shredded paper in location 1, but choose colored beads in location 2. The crucial differences with Experiment 4 were that now both food cups were placed along the same short wall within the rectangular test box (15 cm apart) and animals were always released from the far end of the test box. Consequently, the rats simultaneously faced both choice cups. Rats were trained for 20 days, one session per day, and the group criterion was set to 80%.

### Results

Given that the Hpc3 rats were impaired on the bidirectional go/no-go spatial discrimination (Experiment 8) yet the hippocampal lesions spared the go/no-go spatial task when the rats approached the digging cup from a single direction, i.e., unidirectional (Experiments 5 and 9), it remains possible that the spatial biconditional deficit observed in Cohort1 and Cohort2 (e.g., Experiments 4 and 6) arose from the requirement to approach the digging cups from two directions in each location. Therefore, Cohort3 was tested on a spatial biconditional task where the rats approached the two digging cups from a single direction in each location.

While both the Sham3 and Hpc3 groups showed evidence of acquiring the biconditional discrimination (Fig. 3; main effect of session *F*_(4,76)_ = 40.6, *P* < 0.001; main effect of group *F*_(1,19)_ = 2.85, *P* = 0.108), there was also a significant group × block interaction (*F*_(4, 76)_ = 3.48, *P* = 0.012). This interaction reflected the slower learning by the Hpc3 rats. Consequently, the simple effects showed that the Sham3 group made significantly more correct responses compared with the Hpc3 group on the final two blocks of training (Block 4: *F*_(1,95)_ = 5.65, *P* = 0.02; Block 5: *F*_(1,95)_ = 7.56, *P* = 0.007). In summary, despite the ability of the Hpc3 rats to discriminate the critical locations (Experiment 9), they were impaired when required to use these same locations to solve the biconditional task.

#### Correlations between performance and extent of lesion

Estimates were made of the total extent of hippocampal tissue loss, of dorsal hippocampal tissue loss, and of unintended cortical tissue loss for all three cohorts. Correlations with performance were then examined on those tasks where there was a lesion-induced deficit. No significant correlations (*P* > 0.05) were found for any of these tasks. For Cohort 3 only, there was a significant correlation between extent of dorsal hippocampal tissue loss and cortical tissue loss (*r* = 0.68, *P* = 0.043).

## DISCUSSION

The rodent hippocampus has multiple functions (Moser et al., 1995; Bannerman et al., 1999; O'Mara, 2005; McNaughton, 2006; Fanselow and Dong, 2010). One of the best established of these is in providing a “cognitive map” (O'Keefe and Nadel, 1978). Embedded within this function is the ability to combine patterns of stimuli in unique spatial configurations. This particular ability was examined across three cohorts of rats with hippocampal lesions. While the principal experiments focused on spatial and biconditional learning, a preliminary study showed that the hippocampal lesions severely impaired T-maze alternation (Cohort 1), so confirming the effectiveness of the surgical protocols (Aggleton et al., 1986; Bannerman et al., 1999). The nine subsequent experiments revealed two interlinked sets of problems that required an intact hippocampus, which contrasted with closely related problems that could be solved by rats with hippocampal lesions (Table 2).

One set of problems sensitive to hippocampal damage comprised spatial biconditional learning (Experiments 4, 6, and 10). For these tasks, distal location cues determined in which of two media to dig for food, that is, in one location select medium A, but in the other location select medium B. The hippocampal deficit for the location biconditional task was highly specific as the same rats could distinguish different media (Experiment 2) and different room locations (Experiments 5 and 9). Furthermore, the rats could successfully learn a biconditional task (Experiment 3) when contextual floor and wall cues signaled the correct choice. This intact contextual biconditional performance (Experiment 3) contradicts any general theory that assumes the hippocampus is required for all configural learning, that is, learning when only specific combinations of shared elements signal reinforcement (Rudy and Sutherland, 1995). As the effects of hippocampal lesions on configural learning have proved so variable (McDonald et al., 1997; Coutureau et al., 2002; Sanderson et al., 2006; Saksida et al., 2007; Rudy, 2009), a new perspective is needed.

One potential explanation is that the hippocampus is preferentially engaged by distal cues. Here, “proximal” refers to stimuli, including visual cues that are available by direct exploration, that is, no further than the tip of the nose or the vibrissae (Parron et al., 2004). In contrast, distal cues are beyond the rat's “working space” (Parron et al., 2004). Previous results indicate that the hippocampus is important for distal cues while other regions, for example, parietal cortex, can additionally process proximal cues (Shapiro et al., 1997; Save and Poucet, 2000; Hudon et al., 2003; Renaudineau et al., 2007). Consequently, the variable effects of hippocampal system lesions on conditional and biconditional learning (Gaffan and Harrison, 1989; Jarrard, 1993; McDonald et al., 1997; Deacon et al., 2001; Sanderson et al., 2006) have sometimes been linked to this proximal/distal dimension (Bussey et al., 2000; Deacon et al., 2001; Henry et al., 2004).

There are, however, shortcomings with this proximal/distal distinction. These shortcomings include the ability of the current rats to solve many of the location (“go/no-go”) discriminations, which presumably involved distal cues (Experiments 5 and 9). One refinement might be to narrow the hippocampal requirement to configural learning that involves distal cues. However, rats with hippocampal lesions can successfully relearn biconditional discriminations and transverse patterning problems when distinguishing complex visual cues from a distance (e.g., AB+, CD+, AC−, and BD−; Sanderson et al., 2006; see also Saksida et al., 2007). A further problem is in deciding a priori when cues are “distal” or “proximal” (Good et al., 1998). For example, rats with hippocampal lesions failed to learn a configural task (Iordanova et al., 2009) that involved integrating a specific tone with a particular conditioning box with a particular time of day, yet it is unclear which cue classes are proximal and which are distal.

An alternative explanation (see Rudy, 2009) reflects the fact that the proximal test box cues in the spared biconditional task (Experiment 3) could be readily discriminated by their individual features (different floor colors and textures). In contrast, the distal spatial cues for the other biconditional tasks (Experiments 4, 6, and 10 – all impaired) were heterogeneous and included common cues. The latter feature was presumably also present in the bidirectional go/no-go location discrimination when each location was approached from two opposing directions (Experiment 8, see Fig. 4 upper right). In contrast, rats with hippocampal lesions could discriminate two locations when each was approached from just one direction (“unidirectional”), that is, where the location demands were simpler. Indeed for two of the three unidirectional experiments (Experiments 5,9) the performance of the rats with hippocampal lesions was very similar to that of the controls. It might be argued that the hippocampal deficit on the bidirectional go/no-go task (Experiment 8) reflects a specific loss of direction information (Taube, 2007), as opposed to location learning. While seemingly plausible, the same rats could readily discriminate two directions (Experiments 5 and 9) and any direction strategy would presumably rely on distal visual stimuli (Taube, 2007). Consequently, the go/no-go tasks, which were successfully mastered, still required the discrimination of visual cues beyond the test box.

A potential solution is that the rats with hippocampal lesions could make use of individual features to solve the unidirectional place task and the contextual biconditional problem (since there were no common cues to disambiguate), but failed when required to apply configural learning to situations involving multiple, common cues differentiated by their locations. This distinction, predicted by Rudy (2009) in his two system model of context learning, is exemplified by the finding that monkeys with fornix lesions are impaired on conditional problems when the room stimuli signaling whether to choose item A or B contain common elements, but are unimpaired when the room stimuli to be discriminated contain unique elements (Gaffan and Harrison, 1989; but see Markowska et al., 1989; Dumont et al., 2007).

Previous studies have repeatedly implicated the hippocampus in binding together an item with its location (e.g., Kesner, 1990; Ennaceur et al., 1997; Day et al., 2003; Komorowski et al., 2009; Eichenbaum et al., 2012). This study leads, however, to a more restricted account of location given the sparing when location is determined by local cues that involve no inherent geometry (Experiment 3). The implication is that when the precise spatial (geometric) relationships between critical stimuli are integral to the problem, the task becomes hippocampal dependent. One theoretical framework that captures these issues describes this class of problem as “structural.” This term refers to learning about the geometric relationships or temporal relationships between two or more cues, within the framework of configural learning (George et al., 2001; George and Pearce, 2003). Indeed, it has already been argued that the hippocampus may be required for such “structural learning” (Aggleton and Pearce, 2001; Aggleton et al., 2007). Critically, because structural learning involves the spatial relationships between multiple cues, it can ensure the separation of different scenes, even if they contain common elements, as they will be individually structured, that is, configural learning that binds together common cues in unique geometric (or temporal) ensembles. A prototypical example is the ability to discriminate a scene containing black to the left of white from a scene containing black to the right of white (George et al., 2001). As all elements are shared (black and white) it is not sufficient to learn the configuration of black with white. To solve the discrimination, their relative positions must also be incorporated. By this structural learning account, a rat without a hippocampus should be able to learn to go to a distant location signaled by a black rather than a white wall, or a black and white wall when that information alone is sufficient to identify the wall unambiguously (as there is no structural demand). The same rat should fail, however, when required to bring together cues in unique combinations that involve relative position (Sanderson et al., 2006).

To demonstrate structural learning unambiguously it has proved necessary to train rats on a concurrent series of complex item/place discriminations. For example, learning that white to the left of black is rewarded, but not vice versa (i.e., W|B+ versus B|W−) requires two other concurrent discriminations to ensure reliance on structural information (George et al., 2001; George and Pearce, 2003; Sanderson et al., 2006). Simply training a rat to learn W|B+ versus B|W− risks the confound that a rat which always looks to the left could solve the simpler problem W+ versus B− (a similar elemental solution exists if the rat always looks to the right, B+ versus W−). Even so, hippocampal lesions often impair less formal tests of structural learning such as detecting spatial or temporal rearrangements of stimuli (e.g., Save et al., 1992; Agster et al., 2002; Mumby et al., 2002; Manns and Eichenbaum, 2009; Brown et al., 2010; Barker and Warburton, 2011).

Support for this structural model comes not only from finding that formal tests of structural learning are disrupted by hippocampal lesions (Sanderson et al., 2006), but also by finding that hippocampal lesions can spare configural discriminations, such as biconditional learning and transverse patterning, when they involve visual elements that do not require item-location binding (Gallagher and Holland, 1992; Davidson et al., 1993; Bussey et al., 1998; Sanderson et al., 2006; Saksida et al., 2007). Item-location binding was, however, required in a series of related studies where it was found that hippocampal lesions impair biconditional learning when a specific cue at the choice point of a maze signaled a reward in a particular location (Sziklas and Petrides, 2002) or when one location signaled the selection of object X while a second location signaled the selection of object Y (Dumont et al., 2007). The latter finding is essentially replicated in Experiments 4, 6, and 10, but the current design led to much more rapid task acquisition than that seen in these previous studies (Sziklas and Petrides, 2002; Dumont et al., 2007), making it feasible to test the same rats on related problems (spatial discriminations, nonspatial biconditionals) designed to isolate better the critical task features.

Returning to the present experiments, structural learning would not be required for solving the context biconditional problem (Experiment 3), as this configural task can be solved by learning that particular combinations of stimuli (digging media and floor surfaces) signal the presence or absence of food, without requiring any knowledge of the spatial (or structural) relationships between the two types of cue. Likewise, learning that food could be found in one location, but not another (Experiments 5, 7, and 9) requires neither configural or structural learning, if all that is necessary is to associate individual salient cues in the different locations with the presence or absence of reward. However, when it comes to solving a biconditional discrimination based on two digging media presented in two different locations, then structural learning is required. This task can be solved only by appreciating that food can be found when a particular digging medium is in a particular location. That is, configural learning involving the spatial structure between the specific medium and the various room cues used to define its spatial location. Thus, if the hippocampus is important for structural learning, lesions would be expected to disrupt performance on the last of the foregoing tasks, but not the first two, which is the pattern of results that we found. Damage to the hippocampus also disrupted performance on the spatial go/no-go task, in which a single digging medium occupied two different locations, each in two different regions of the test arena (Experiment 8). Here, it is potentially difficult to associate a particular test box location with food because (i) no location was always paired with food (as on some trials there would be no cup in that location) and (ii) the bidirectional testing would encourage the processing of common spatial cues in many trials, whether they were rewarded or unrewarded (see Fig. 4 upper right). Instead, to find food on every trial the optimal strategy is to learn about the spatial disposition of the cues that surround the four cup locations, and their position with respect to the digging cup That is, the optimal solution of the problem required structural learning.

At first sight, the present results also fit the notion that the hippocampus, in particular the dentate gyrus, is required for effective pattern separation (Gilbert et al., 1998; Leutgeb et al., 2007). According to this viewpoint, loss of the hippocampus would impair the ability to discriminate locations with common, overlapping cues and so contribute to the deficits in both the bidirectional go/no-go discrimination (Experiment 8) and the location biconditional tasks (Experiments 4 and 6). The same account (pattern separation) would also explain the intact contextual biconditional learning (Experiment 3) as each test box contains salient unique features. While this alternative account appears attractive, it might not predict the biconditional learning deficit in Experiment 10 as the rats could already discriminate the locations (Experiment 9), yet were still impaired. Here, the structural account would argue that by adding the two digging cups in each location the normal animal acquires the unique spatial configuration of the correct cup with its distal cues as this added information better distinguishes the particular cup when it is in two places (see also Dumont et al., 2007, condition 2). The fact that this structural element is not a necessity in Experiment 10 may also explain the ability of the hippocampectomized rats to partially learn this problem, albeit inefficiently. A final problem with the pattern separation hypothesis is in predicting a priori when pattern separation is required, for example, why hippocampal lesions disrupt only some configural tasks involving visual stimuli (Sanderson et al., 2006; see also Iordanova et al., 2011).

In summary, hippocampal lesions disrupted configural learning involving spatial cues and location discriminations when rats were exposed to overlapping spatial cues. To combine these two categories of problems, it is supposed that similar binding functions bring together specific cues in unique spatial arrangements (for location discriminations) and bring together specific items (digging media) with particular spatial cues to create unique scenes that can enter into associations (biconditional discrimination). Both functions require structural learning. This form of learning could then prove integral to creating cognitive maps, as place cells are often not tied to individual cues or direction (O'Keefe, 1979; Muller et al., 1994). Furthermore, hippocampal activity is sensitive to combinations of cue and location, such that global remapping follows the presentation of familiar cues in changed places (Leutgeb et al., 2005). Indeed, units responsive to item-place conjunctions are found in the hippocampus when rats perform biconditional tasks of the sort used in this study (Komorowski et al., 2009). Consequently, structural learning should prove particularly valuable when discriminating arrays with common elements but individual spatial configurations (Gaffan and Harrison, 1989; Holland and Bouton, 1999; Moses et al., 2007), so helping the representation of scenes distinguished by time and space (Gaffan, 1994; Eichenbaum, 2001; Hassabis and Maguire, 2007) and explaining perceptual deficits ascribed to the hippocampus (Lee et al., 2012).

## References

[b1] Aggleton JP, Pearce JM (2001). Neural systems underlying episodic memory: Insights from animal research. Philos Trans Roy Soc.

[b2] Aggleton JP, Hunt PR, Rawlins JNP (1986). The effects of hippocampal lesions upon spatial and nonspatial tests of working memory. Behav Brain Res.

[b3] Aggleton JP, Sanderson DJ, Pearce JM (2007). Structural learning and the hippocampus. Hippocampus.

[b4] Agster KL, Fortin NJ, Eichenbaum H (2002). The hippocampus and disambiguation of overlapping sequences. J Neurosci.

[b5] Bannerman DM, Yee BK, Good MA, Heupel MJ, Iversenm SD, Rawlins JNP (1999). Double dissociation of function within the hippocampus: A comparison of dorsal, ventral, and complete hippocampal cytotoxic lesions. Behav Neurosci.

[b6] Barker GRI, Warburton EC (2011). When is the hippocampus involved in recognition memory?. J Neurosci.

[b7] Brown MW, Warburton EC, Aggleton JP (2010). Recognition memory: Material, processes, and substrates. Hippocampus.

[b8] Bussey TJ, Warburton EC, Aggleton JP, Muir JL (1998). Fornix lesions can facilitate acquisition of the transverse patterning task: A challenge for “configural” theories of hippocampal function. J Neurosci.

[b9] Bussey TJ, Duck JD, Muir JL, Aggleton JP (2000). Distinct patterns of behavioural impairments resulting from fornix transection or neurotoxic lesions of the perirhinal and postrhinal cortices in the rat. Behav Brain Res.

[b10] Coutureau E, Killcross AS, Good M, Marshall VJ, Ward-Robinson J, Honey RC (2002). Acquired equivalence and distinctiveness of cues: II. Neural manipulations and their implications. J Exp Psych: Anim Behav Process.

[b11] Davidson TL, McKernan MG, Jarrard LE (1993). Hippocampal lesions do not impair negative patterning: A challenge to configural association theory. Behav Neurosci.

[b12] Day M, Langston R, Morris RGM (2003). Glutamate-receptor-mediated encoding and retrieval of paired-associate learning. Nature.

[b13] Deacon RMJ, Bannerman DM, Rawlins JNP (2001). Conditional discriminations based on external and internal cues in rats with cytotoxic hippocampal lesions. Behav Neurosci.

[b14] Dumont J, Petrides M, Sziklas V (2007). Functional dissociation between fornix and hippocampus in spatial conditional learning. Hippocampus.

[b15] Eichenbaum H (2001). The hippocampus and declarative memory: cognitive mechanisms and neural codes. Behav Brain Res.

[b16] Eichenbaum H (2004). Hippocampus: Cognitive processes and neural representations that underlie declarative memory. Neuron.

[b17] Eichenbaum H, Sauvage M, Fortin N, Komorowski R, Lipton P (2012). Towards a functional organization of episodic memory in the medial temporal lobe. Neurosci Biobehav Rev.

[b18] Ennaceur A, Neave NJ, Aggleton JP (1997). Spontaneous object recognition and object location memory in rats: The effects of lesions in the cingulate cortices, the medial prefrontal cortex, the cingulum bundle and the fornix. Exp Brain Res.

[b19] Fanselow MS, Dong H-W (2010). Are the dorsal and ventral hippocampus functionally distinct structures?. Neuron.

[b20] Gaffan D (1994). Scene-specific memory for objects: A model of episodic memory impairment in monkeys with fornix transaction. J Cog Neurosci.

[b21] Gaffan D, Harrison S (1989). Place memory and scene memory: Effects fornix transection in the monkey. Exp Brain Res.

[b22] Gallagher M, Holland PC (1992). Preserved configural learning and spatial learning impairment in rats with hippocampal damage. Hippocampus.

[b23] George DN, Pearce JM (2003). Discrimination of structure: II. Feature binding. J Exp Psych: Anim Behav Process.

[b24] George DN, Ward-Robinson J, Pearce JM (2001). Discrimination of structure: I. Implications for connectionist theories of discrimination learning. J Exp Psych: Anim Behav Process.

[b25] Gilbert PE, Kesner RK, DeCoteau WE (1998). Memory for spatial location: Role of the hippocampus in mediating spatial pattern separation. J Neurosci.

[b26] Good M, de Hoz L, Morris RGM (1998). Contingent versus incidental context processing during conditioning: Dissociation after excitotoxic hippocampal plus dentate gyrus lesions. Hippocampus.

[b27] Hassabis D, Maguire EA (2007). Deconstructing episodic memory with construction. Trends Cogn Sci.

[b28] Henry J, Petrides M, St-Laurent M, Sziklas V (2004). Spatial conditional associative learning: Effects of thalamo-hippocampal disconnections in rats. Neuroreport.

[b29] Holland PC, Bouton ME (1999). Hippocampus and context in classical conditioning. Curr Opin Neurobiol.

[b30] Howell DC (1982). Statistical Methods for Psychology.

[b31] Hudon C, Dore FY, Goulet S (2003). Impaired performance of fornix-transected rats on a distal, but not on a proximal, version of the radial-arm maze cue task. Behav Neurosci.

[b32] Iordanova MD, Burnett D, Good M, Aggleton JP, Honey RC (2009). The role of the hippocampus in mnemonic integration and retrieval: Complementary evidence from lesion and inactivation studies. Eur J Neurosci.

[b33] Iordanova MD, Burnett D, Good M, Honey RC (2011). Pattern memory involves both elemental and configural processes: Evidence from the effects of hippocampal lesions. Behav Neurosci.

[b34] Jarrard LE (1993). On the role of the hippocampus in learning and memory in the rat. Behav Neural Biol.

[b35] Kesner RP, Kesner RP, Olton DS (1990). Learning and memory in rats with an emphasis on the hippocampal formation. Neurobiology of comparative cognition.

[b36] Komorowski RW, Manns JR, Eichenbaum H (2009). Robust conjunctive item-place coding by hippocampal neurons parallels learning what happens where. J Neurosci.

[b37] Lee ACH, Yeung L-K, Barense MD (2012). The hippocampus and visual perception. Front Hum Neurosci.

[b38] Leutgeb S, Leutgeb JK, Barnes CA, Moser EI, McNaughton BL, Moser M-B (2005). Independent codes for spatial and episodic memory in hippocampal neuronal ensembles. Science.

[b39] Leutgeb JK, Leutgeb S, Moser M-B, Moser EI (2007). Pattern separation in the dentate gyrus and CA3 of the hippocampus. Science.

[b40] Manns JR, Eichenbaum H (2009). A cognitive map for object memory in the hippocampus. Learn Mem.

[b41] Markowska AL, Olton DS, Murray EA, Gaffan D (1989). A comparative analysis of the role of fornix and cingulate cortex in memory: rats. Exp Brain Res.

[b42] McDonald RJ, Murphy RA, Guarraci FA, Gortler JR, White NM, Baker AG (1997). Systematic comparison of the effects of hippocampal and fornix-fimbria lesions on the acquisition of three configural discriminations. Hippocampus.

[b43] McNaughton N (2006). The role of the subiculum within the behavioural inhibition system. Behav Brain Res.

[b44] Morris RGM, Garrud P, Rawlins JNP, O'Keefe J (1982). Place navigation impaired in rats with hippocampal lesions. Nature.

[b45] Moser EI, Kropff E, Moser M-B (2008). Place cells, grid cells, and the brain's spatial representation system. Ann Rev Neurosci.

[b46] Moser M-B, Moser EI, Forrest E, Andersen P, Morris RGM (1995). Spatial learning with a minislab in the dorsal hippocampus. Proc Natl Acad Sci USA.

[b47] Moses SN, Winocur G, Ryan JD, Moscovitch M (2007). Environmental complexity affects contextual fear conditioning following hippocampal lesions in rats. Hippocampus.

[b48] Muller RU, Bostock E, Yaube JS, Kubie JL (1994). On the directional properties of hippocampal place cells. J Neurosci.

[b49] Mumby DG, Gaskin S, Glenn MJ, Schramek TE, Lehman H (2002). Hippocampal damage and exploratory preferences in rats: Memory for objects, places, and contexts. Learn Mem.

[b50] O'Keefe J (1979). A review of the hippocampal place cells. Progr Neurobiol.

[b51] O'Keefe J, Burgess N (1996). Geometric determinants of the place fields of hippocampal neurons. Nature.

[b52] O'Keefe J, Nadel L (1978). The hippocampus as a cognitive map.

[b53] O'Mara SM (2005). The subiculum; what it does, what it might do, and what neuroanatomy has yet to tell us. J Anat.

[b54] Parron C, Poucet B, Save E (2004). Entorhinal cortex lesions impair the use of distal but not proximal landmarks during place navigation in the rat. Behav Brain Res.

[b55] Paxinos G, Watson C (2005). The Rat Brain in Stereotaxic Coordinates.

[b56] Pearce JM, Roberts AD, Good M (1998). Hippocampal lesions disrupt navigation based on cognitive maps but not heading vectors. Nature.

[b57] Renaudineau S, Poucet B, Save E (2007). Flexible use of proximal objects and distal cues by hippocampal place cells. Hippocampus.

[b58] Rudy JW (2009). Context representations, context functions, and the parahippocampal-hippocampal system. Learn Mem.

[b59] Rudy JW, Sutherland RJ (1995). Configural association theory and the hippocampal formation: An appraisal and reconfiguration. Hippocampus.

[b60] Saksida LM, Bussey TJ, Buckmaster CA, Murray EA (2007). Impairment and facilitation of transverse patterning after lesions of the perirhinal cortex and hippocampus, respectively. Cereb Cortex.

[b61] Sanderson D, Pearce JM, Kyd R, Aggleton JP (2006). The importance of the rat hippocampus for learning the structure of visual arrays. Eur J Neurosci.

[b62] Save E, Poucet B (2000). Involvement of the hippocampus and associative parietal cortex in the use of proximal and distal landmarks for navigation. Behav Brain Res.

[b63] Save E, Poucet B, Foreman N, Buhot N (1992). Object exploration and reactions to spatial and non-spatial changes in hooded rats following damage to parietal cortex or hippocampal formation. Behav Neurosci.

[b64] Shapiro ML, Tanila H, Eichenbaum H (1997). Cues the hippocampal place cells encode: Dynamic and hierarchical representation of local and distal stimuli. Hippocampus.

[b65] Sutherland RJ, Rudy JW (1989). Configural association theory: The role of the hippocampal formation in learning, memory and amnesia. Psychobiology.

[b66] Sziklas V, Petrides M (2002). Effects of lesions to the hippocampus or the fornix on allocentric conditional associative learning in rats. Hippocampus.

[b67] Sziklas V, Lebel S, Petrides M (1998). Conditional associative learning and the hippocampal system. Hippocampus.

[b68] Taube JS (2007). The head direction signal: origins and sensory motor integration. Ann Rev Neurosci.

